# Low-level regulatory T-cell activity is essential for functional type-2 effector immunity to expel gastrointestinal helminths

**DOI:** 10.1038/mi.2015.73

**Published:** 2015-08-19

**Authors:** K A Smith, K J Filbey, L A Reynolds, J P Hewitson, Y Harcus, L Boon, T Sparwasser, G Hämmerling, R M Maizels

**Affiliations:** 1 Institute of Immunology and Infection Research, and Centre for Immunity, Infection and Evolution, University of Edinburgh, Edinburgh, UK; 2 Bioceros Holding BV, Utrecht, The Netherlands; 3 TwinCore, Hannover, Germany; 4 Division of Molecular Immunology, German Cancer Research Center, Heidelberg, Germany

## Abstract

Helminth infection is frequently associated with the expansion of regulatory T cells (Tregs) and suppression of immune responses to bystander antigens. We show that infection of mice with the chronic gastrointestinal helminth *Heligmosomoides polygyrus* drives rapid polyclonal expansion of Foxp3^+^Helios^+^CD4^+^ thymic (t)Tregs in the lamina propria and mesenteric lymph nodes while Foxp3^+^Helios^−^CD4^+^ peripheral (p)Treg expand more slowly. Notably, in partially resistant BALB/c mice parasite survival positively correlates with Foxp3^+^Helios^+^CD4^+^ tTreg numbers. Boosting of Foxp3^+^Helios^+^CD4^+^ tTreg populations by administration of recombinant interleukin-2 (rIL-2):anti-IL-2 (IL-2C) complex increased worm persistence by diminishing type-2 responsiveness *in vivo*, including suppression of alternatively activated macrophage and granulomatous responses at the sites of infection. IL-2C also increased innate lymphoid cell (ILC) numbers, indicating that Treg functions dominate over ILC effects in this setting. Surprisingly, complete removal of Tregs in transgenic Foxp3-DTR mice also resulted in increased worm burdens, with “immunological chaos” evident in high levels of the pro-inflammatory cytokines IL-6 and interferon-γ. In contrast, worm clearance could be induced by anti-CD25 antibody–mediated partial depletion of early Treg, alongside increased T helper type 2 responses and without incurring pathology. These findings highlight the overarching importance of the early Treg response to infection and the non-linear association between inflammation and the prevailing Treg frequency.

## Introduction

Foxp3-expressing CD4^+^ regulatory T cells (Tregs) reside within the tissue and lymphatics at steady state and have a fundamental role in the control of immune reactivity and protection from autoimmune disease^1, 2, 3^ as well as in the responsiveness to commensals at the mucosa.^4, 5, 6^ Although Treg functions may decline in many acute inflammatory settings,^7^ they frequently appear to be expanded after challenge with chronic viral and helminth pathogens.^8, 9^ However, in most instances it is not clear whether Treg expansion represents a homeostatic response to control aggravated antipathogen effector mechanisms or is driven by the pathogens themselves as a strategy to prolong infection. Importantly, this distinction determines whether intervention to dampen Treg activity would promote immunity or exacerbate pathology.

Colonization with the chronic intestinal helminth *Heligmosomoides polygyrus* is associated with the expansion of CD4^+^Foxp3^+^ T cells within fully susceptible (C57BL/6) and partially resistant (BALB/c) strains of mice early in response to infection in the lamina propria (LP) and mesenteric lymph nodes (MLNs).^10, 11^ Treg expansion appears to be promoted by the parasite through its release of a transforming growth factor-β-like ligand^12^ and depends on the expression of ICOS (inducible T-cell costimulator) on host T cells.^13^ Furthermore, our recent results suggest that aberrant Treg phenotypes early in infection are associated with enhanced t helper type 2 (Th2) responsiveness and increased parasite expulsion in mice deficient in interleukin (IL)-6.^14^

Antibody (Ab)-mediated depletion of CD25^+^ Tregs was first shown to significantly reduce the number of adult parasites when administered to infected mice in a permissive model of filariasis, contingent on co-administration of Abs to GITR (glucocorticoid-induced tumor necrosis factor receptor–related) or CTLA-4 (cytotoxic T-lymphocyte-associated protein 4).^15, 16^ Subsequently, predepletion of thymic Tregs early in infection was shown to heighten immunity.^17^ Likewise, depletion of Tregs during patency of the parasitic trematode *Schistosoma mansoni* using anti-CD25 Ab or a genetically modified mouse model (DEREG) also decreased parasite egg numbers by elevating the schistosome-specific Th2 response.^18, 19^ However, in infections with specific chronic isolates of *Trichuris muris*, Treg depletion using anti-CD25 Ab primarily exacerbated host pathology rather than elicited protective immunity,^20^ although parasite burdens were reduced following anti-GITR treatment.^20^ *T. muris* parasite burden was also reduced through early depletion of Foxp3^+^ T cells in Foxp3-DTR mice;^21^ however, it was unaffected through early depletion of Foxp3^+^ T cells in DEREG mice.^22^ It was also reported that Treg depletion of Foxp3-DTR C57BL/6 DEREG mice did not alter *H. polygyrus* worm burden 14 days postinfection,^23^ although this time point is before even genetically resistant SJL mice begin to expel parasites.^24^

Because the kinetic and genetic contexts of infection are emerging as key determinants of Treg activity in helminth infection,^21, 24^ we have investigated the effects of Treg manipulation on the course of *H. polygyrus* infection in a range of settings. We not only make use of recombinant IL-2:anti-IL-2 complexes (IL-2C) to boost thymic-derived Treg populations *in vivo* prior to infection of BALB/c mice but also adopt two strategies for Treg depletion in both BALB/c and C57BL/6 genetic backgrounds, through the use of transgenic DEREG^25^ and Foxp3.LuciDTR mice.^26^ These tools permitted us to assess the impact of Treg depletion to differing degrees, at different stages of infection, and in contrasting genetic strains.

As reported below, boosting of thymic-derived Treg populations *in vivo* using IL-2C prior to *H. polygyrus* infection inhibited innate and adaptive type-2 responses and ablated adult worm expulsion in more resistant BALB/c mice, despite also increasing innate lymphoid cell (ILC) numbers. Interestingly, a more complex, mixed inflammatory response dominated by pro-inflammatory Th1 cytokines emerged in Treg-depleted transgenic BALB/c Foxp3.LuciDTR mice. Reflecting this immune-skewing, parasite immunity was compromised and worm burdens increased. Complete depletion of Treg in both Foxp3.LuciDTR and DEREG mice at differing time points postinfection resulted in significant pathology, including weight loss, and reversal of the partial resistance of BALB/c mice. In contrast, partial but incomplete early Treg depletion with anti-CD25 Abs in infected BALB/c mice resulted in increased adaptive type-2 responses and increased worm expulsion, without significantly altering innate type-2 immunity. Hence, optimal type 2 immunity requires a low level of regulatory activity from Foxp3^+^ T cells.

## RESULTS

### Expansion of thymic Tregs in H. polygyrus infection

**Figure 1:**
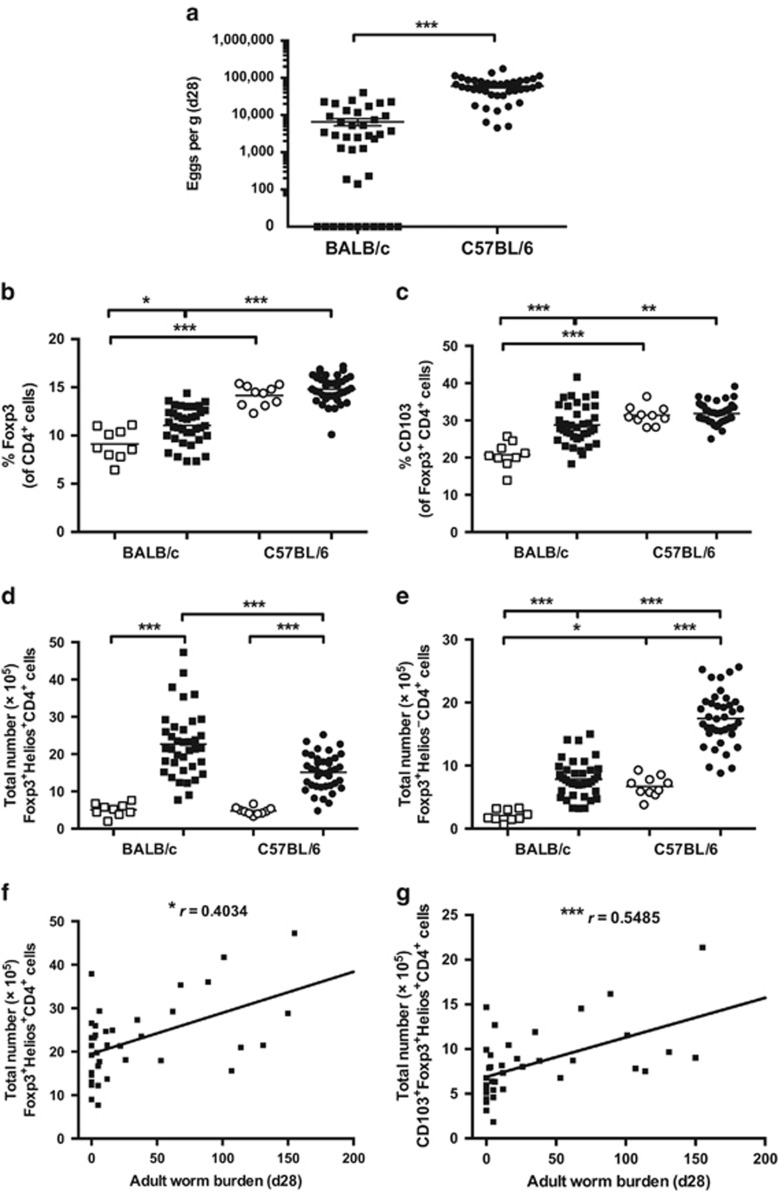
Differential susceptibility to infection and generation of thymic regulatory T cells (tTregs). (**a**) Female BALB/c and C57BL/6 mice were infected with 200 L3 stage *H. polygyrus* and adult egg counts were quantified at day 28 postinfection. Single-cell suspensions of mesenteric lymph node were analyzed in naive (white symbols) and day-28 infected (black symbols) mice for the proportion of CD4^+^ T cells expressing (**b**) Foxp3 and (**c**) the percentage of CD4^+^Foxp3^+^ T cells expressing CD103 by flow cytometry. (**d**, **e**) The total numbers of CD4^+^Foxp3^+^ tTregs (Helios^+^) and peripheral Tregs (Helios^−^) were determined in the two strains. (**f**, **g**) Significant positive correlations between adult worm burdens and total numbers of tTregs and CD103^+^ tTregs were found in BALB/c mice; *r*=Spearman *r* value. Data shown include totals of 10 naive and 40 infected mice of each strain, as previously detailed.^31^

Infection with the intestinal helminth parasite *H. polygyrus* is associated with the expansion of regulatory CD4^+^ T-cell populations within the MLN and LP as the parasite establishes a chronic infection.^10, 11, 12, 27, 28^ Moreover, Tregs from *H. polygyrus*–infected mice show enhanced regulatory function *in vitro*.^10, 11^ As susceptibility to *H. polygyrus* infection,^29, 30^ and the degree of Treg expansion,^24^ varies between genetic backgrounds of mice, we compared Treg populations in partially resistant BALB/c mice and fully susceptible C57BL/6 mice. As previously reported, by day 28 postinfection, BALB/c mice harbor far fewer adult worms^31^ and produce many less fecal eggs (Figure 1a) than C57BL/6 animals, with some individuals spontaneously clearing infection. Within the MLN, infection of BALB/c mice drove increased Foxp3^+^ Treg frequency, while C57BL/6 mice had constitutively high levels, which did not rise significantly following infection (Figure 1b). Similarly, a significant induction of CD103, considered an activation marker within the mucosal Treg compartment,^32^ was observed in BALB/c mice while expression was constitutively higher in the more susceptible C57BL/6 mouse (Figure 1c).

Helios and Neuropilin-1 expression both serve as specific markers for thymic-derived natural Treg.^33, 34, 35^ Following confirmation that Neuropilin-1 expression strongly correlated with Helios expression in CD4^+^Foxp3^+^ T cells (94.8%±1.1% of Helios^+^ cells also expressed Neuropilin-1), we next compared the relative induction of thymic (tTreg) and peripheral (pTreg) cells, as indicated by the expression of transcription factor Helios, Interestingly, Helios^+^ tTregs were markedly expanded in infected BALB/c mice (Figure 1d), while C57BL/6 mice show much stronger expansion of Helios^−^ pTregs (Figure 1e). Moreover, in BALB/c mice, the level of infection measured by adult worm burdens at day 28 positively correlated with the number of Helios^+^ tTregs (Figure 1f) but not with numbers of Helios^−^ pTregs (data not shown), and there was an even stronger positive correlation with the number of CD103^+^Helios^+^ tTregs (Figure 1g).

**Figure 2:**
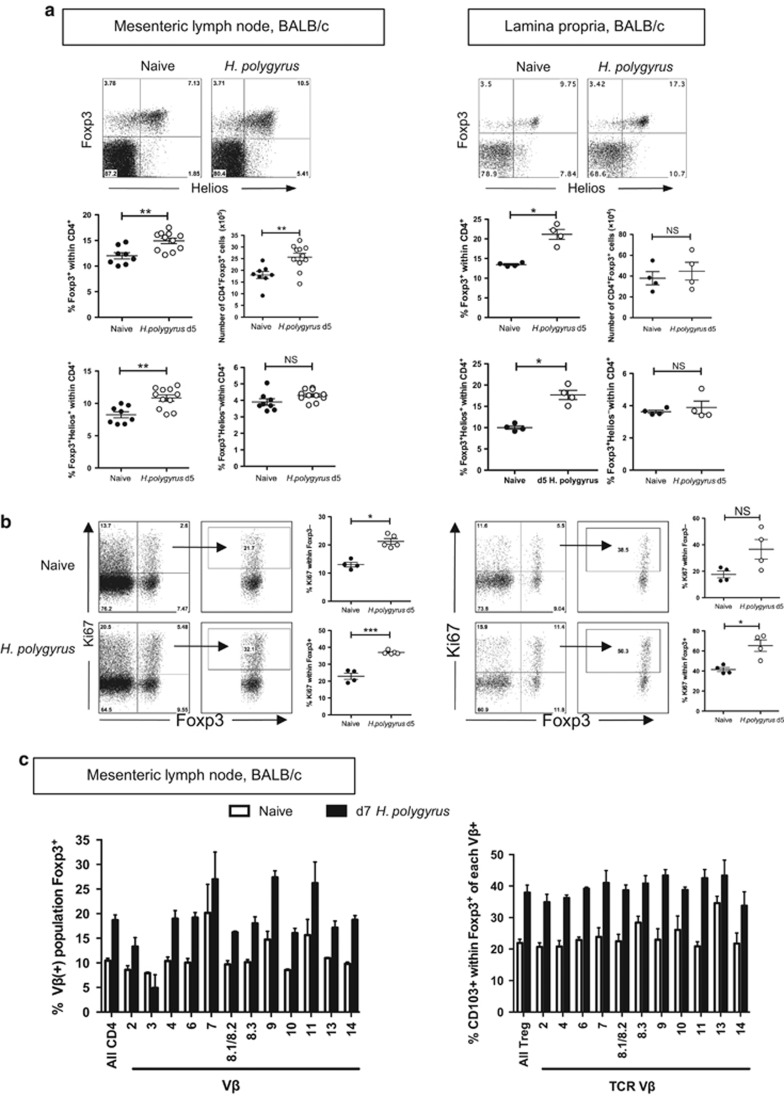
Expansion of Foxp3^+^ regulatory T cells (Tregs) in mesenteric lymph node (MLN) and lamina propria (LP) following *H. polygyrus* infection. (**a**) Female BALB/c mice were naive or infected with 200 L3 stage *H. polygyrus* and single-cell suspensions of (**a** and **b**, left) MLN and (**a** and **b**, right) LP were analyzed by flow cytometry at day 5 postinfection for the proportion and number of Foxp3^+^CD4^+^ Treg and the proportion of Foxp3^+^CD4^+^Helios^+^ thymic Treg and Foxp3^+^CD4^+^Helios^−^ peripheral Treg. (**b**) Proliferation within CD4^+^Foxp3^−^ T-cell and CD4^+^Foxp3^+^ Treg compartments was measured using Ki67^+^. Cells were gated on live, CD4^+^ T cells; fold proliferation was calculated using: mean percentage of Ki67 from infected mice/mean percentage of Ki67 from naive mice. (**c**) T-cell receptor Vβ expression was quantified by flow cytometry within CD4^+^Foxp3^+^ (left) and CD4^+^Foxp3^+^CD103^+^ (right) T cells in naive and day-7 *H. polygyrus*–infected mice. Experiments shown are representative of two experiments with *n*⩾4 mice/group (**a**–**c**). NS, not significant.

To ascertain whether Treg expansion in mucosal tissues is a consequence of chronic infection, or is induced soon after parasite entry, we studied MLN and LP populations at day 5 following infection of BALB/c mice with *H. polygyrus*. At this time point, a significant increase in the proportion of CD4^+^Foxp3^+^ T cells was observed at both sites, as well as an expanded number of CD4^+^Foxp3^+^ T cells within the MLN; moreover, this increment was mirrored in extent within the Foxp3^+^Helios^+^ and not the Foxp3^+^Helios^−^ subset (Figure 2a). We also assessed the proliferation of CD4^+^Foxp3^+^ Treg and CD4^+^Foxp3^−^ T cell populations in the MLN and LP at day 5 postinfection (Figure 2b). Most notably, Treg proliferation was robustly stimulated at both sites and in the LP was more consistently uplifted than was the case for the CD4^+^Foxp3^−^ subset. These data suggest that tTregs respond rapidly and are able to predominate at gastrointestinal mucosal surfaces following *H. polygyrus* infection.^36^ In addition, we profiled the distribution of TCR Vβ expression within Tregs in naive and infected mice; among almost all Vβ types, there was an increase in the proportions of CD4^+^ T cells expressing Foxp3 and CD103 following infection (Figure 2c). Hence, from the early stages of infection there is activation and preferential expansion of Tregs across a broad polyclonal spectrum.

### Enhancement of Treg activation prolongs helminth infection

**Figure 3:**
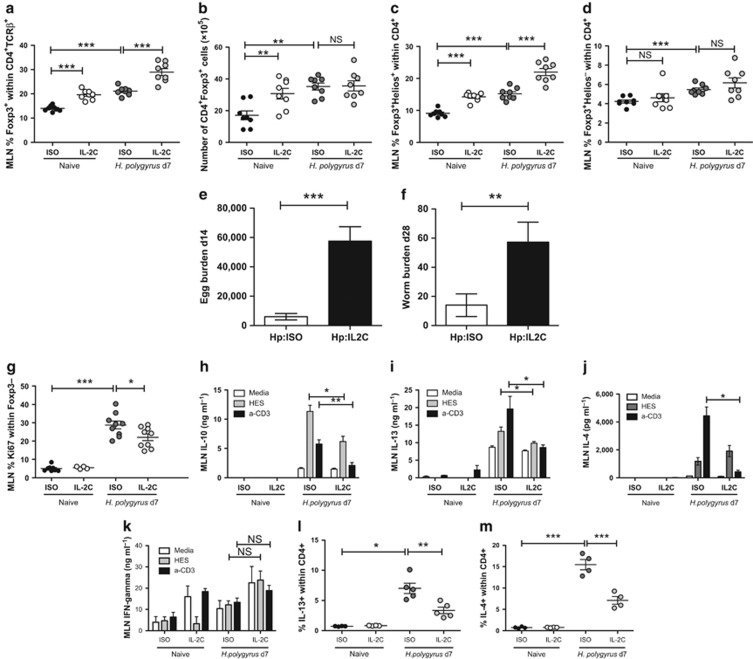
Boosting regulatory T cell (Treg) populations *in vivo* reduces parasite immunity in more resistant BALB/c mice. Naive BALB/c mice were treated with a complex of 2.5 μg recombinant interleukin-2 (rIL-2) and 25 μg anti-IL-2 (IL-2C) or 25 μg rat IgG2a isotype control (ISO) immediately before infection with 200 *H. polygyrus* by gavage. Mice were harvested at day 7 postinfection, and single-cell suspensions of mesenteric lymph node (MLN) were analyzed for (**a**) Foxp3^+^ proportion and (**b**) Foxp3 number, as well as (**c**) thymic Treg and (**d**) peripheral Treg proportions within CD4^+^ cells by flow cytometry. (**e**) Fecal egg burden was determined at day 14 and (**f**) adult worm burden enumerated at day 28 postinfection. MLN from day 7 postinfection were also analyzed for (**g**) the percentage of proliferating (Ki67^+^) effector CD4^+^ T cells or plated and re-stimulated with 1 μg *H. polygyrus* excretory/secretory antigen (HES), anti-CD3, or media for 72 h and production of (**h**) IL-10, (**i**) IL-13, (**j**) IL-4, and (**k**) interferon (IFN)-γ in the supernatant was assessed by enzyme-linked immunosorbent assay. Intracellular straining of ionomycin and phorbol myristate acetate–stimulated CD4^+^ T cells for (**l**) IL-13 and (**m**) IL-4 of the MLN was also performed. Experiments shown are one representative of two experiments with *n*⩾4 mice/group (**a**–**d** and **g**–**m**) or are pooled data from two experiments with *n*⩾4 mice/group (**e**, **f**). NS, not significant.

To determine whether the initial Treg:Teff balance has a crucial role in the outcome of infection, we adopted a strategy to experimentally modulate this balance *in vivo*, thereby analyzing the impact of early expansion of Treg on parasite immunity and the inflammatory response to infection. Using a complex of rIL-2–anti-IL-2 (IL-2C), previously shown to selectively stimulate CD4^+^ Tregs *in vivo*,^37^ the CD4^+^Foxp3^+^ Treg population was boosted immediately before *H. polygyrus* infection of partially resistant BALB/c mice. Injection of a single dose of IL-2C significantly increased the percentage and number of MLN Foxp3^+^CD4^+^ Treg cells in naive mice and further significantly elevated the percentage of MLN Foxp3^+^CD4^+^ Treg in day 7 *H. polygyrus*–infected mice over that identified in infected mice treated with an isotype control (Figure 3a,b). This increase in Treg in response to IL-2C corresponded to an increase in Helios^+^ tTregs but not Helios^−^ pTregs, whereas *H. polygyrus* infection elicited a significant increase in both subsets (Figure 3c,d).

Following administration of IL-2C to BALB/c mice, parasite immunity was significantly impaired, with enhanced egg and adult worm burdens in treated mice at days 14 and 28 postinfection, respectively (Figure 3e,f), converting these animals to a more susceptible status akin to the C57BL/6 strain (Figure 1a,b). Effector CD4^+^ T-cell proliferation was significantly reduced in *H. polygyrus*–infected mice following administration of IL-2C (Figure 3g), which correlated with a reduction in antigen-specific IL-10 and IL-13 (Figure 3h,i) and total IL-4 cytokine production (Figure 3j). Levels of antigen-specific and total interferon (IFN)-γ were not significantly altered by IL-2C administration (Figure 3k). The major source of the type-2 cytokines IL-13 and IL-4 in *H. polygyrus*–infected mice was CD4^+^ T cells, which was significantly reduced following administration of IL-2C (Figure 3l,m).

**Figure 4:**
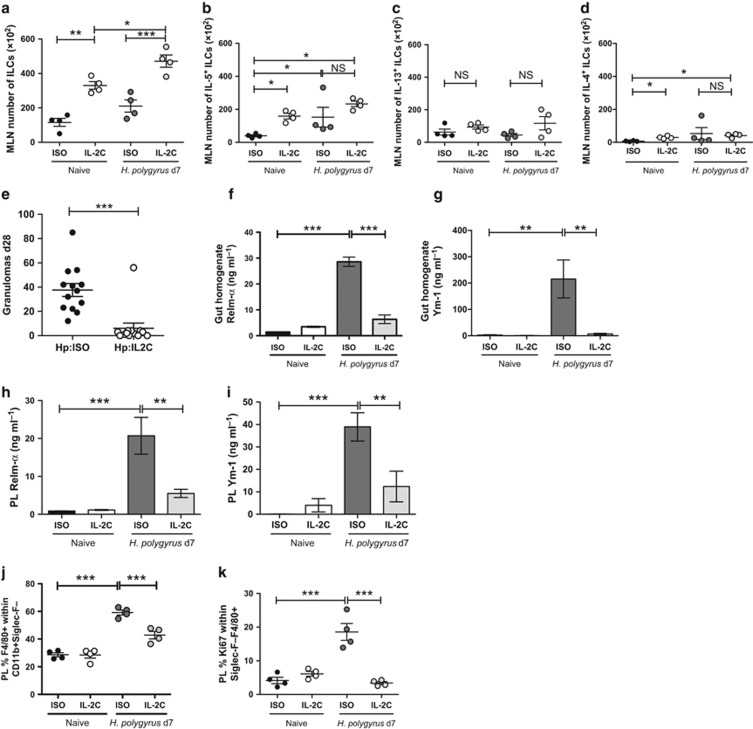
Boosting regulatory T cell populations *in vivo* modifies innate type-2 immunity following *H. polygyrus* infection. Naive BALB/c mice were treated with a complex of 2.5 μg recombinant interleukin-2 (rIL-2) and 25 μg anti-IL-2 (IL-2C) or 25 μg rat IgG2a isotype control (ISO) immediately before infection with 200 *H. polygyrus* by gavage. (**a**) The number of CD3^−^CD4^−^CD8^−^CD5^−^CD11c^−^CD49b^−^F4/80^−^ (Lin)^−^ICOS^+^ innate lymphoid cells (ILCs) expressing (**b**) IL-5, (**c**) IL-13, and (**d**) IL-4 within the mesenteric lymph node (MLN) was also determined at day 7 postinfection by flow cytometry. (**e**) Intestinal granuloma formation was enumerated at day 28 postinfection. The production of the alternative activation markers RELM-α and Ym-1 within (**f**–**i**) the gut homogenate peritoneal lavage (PL) and (**j**) the proportion of macrophages (F4/80^+^ within CD11b^+^Siglec-F^−^) and (**k**) macrophage proliferation (Ki67^+^) within the PL was determined by enzyme-linked immunosorbent assay and flow cytometry at day 7 postinfection. Experiments shown are one representative of two experiments with *n*⩾4 mice/group (**a**–**d** and **f**–**k**) or are pooled data from two experiments with *n*⩾4 mice/group (**e**). NS, not significant.

*H. polygyrus* stimulates strong innate immune responses, including ILC expansion in BALB/c mice,^24^ and extensive alternative activation of macrophages linked to the formation of granulomas.^24, 38^ Somewhat surprisingly, despite the decreased resistance of IL-2C-treated mice to *H. polygyrus*, innate lymphoid populations were significantly increased in naive and infected IL-2C-treated mice (Figure 4a), suggesting that this population cannot influence parasite burden early in infection. The dominant population of ILCs elicited following *H. polygyrus* infection produced IL-5, as determined by intracellular cytokine staining (Figure 4b). The number of IL-5^+^, IL-13^+^, or IL-4^+^ ILCs was not significantly altered by IL-2C administration to *H. polygyrus*–infected mice (Figure 4b–d). We also noted a marked reduction in the number of intestinal granulomas present in IL-2C-treated infected mice (Figure 4e). Analysis of protein production within the gut tissue and peritoneal lavage (PL) revealed that IL-2C treatment significantly reduced the levels of RELM-α and Ym-1, two key products associated with alternatively activated macrophages (Figure 4f–i). IL-2C treatment also significantly reduced the proportion of macrophages within the PL of *H. polygyrus*–infected mice (Figure 4j), and completely ablated their proliferation (Figure 4k). These data demonstrate that early changes in the proportions of Treg can influence both innate and adaptive type-2 responses following helminth infection and directly impact on parasite persistence.

### Immune disruption in infected, Treg-depleted BALB/c mice

**Figure 5:**
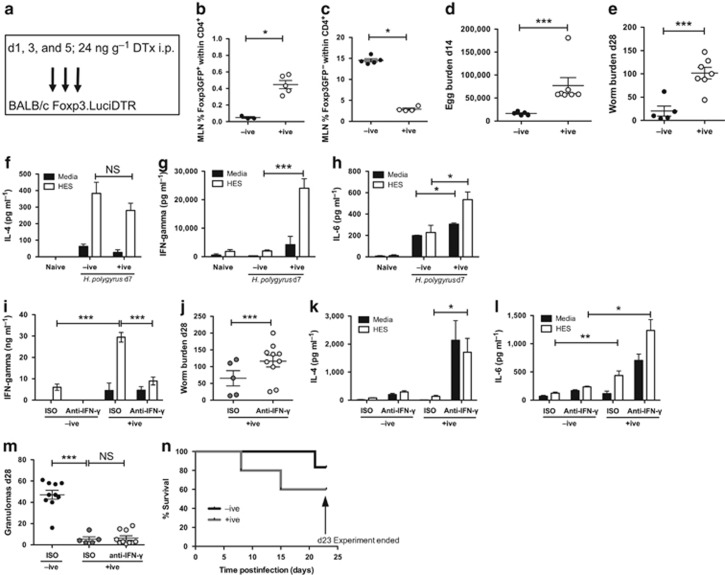
Early depletion of regulatory T cell (Treg) populations *in vivo* transgenic BALB/c Foxp3.LuciDTR mice. (**a**) BALB/c Foxp3.LuciDTR mice were treated with 24 ng g^−1^ diphtheria toxin (DTx) following infection with 200 *H. polygyrus* by gavage. The proportion of (**b**) Foxp3GFP^+^ Treg and (**c**) CD4^+^Foxp3^+^ Treg within the mesenteric lymph node (MLN) of DTx-treated day 7 *H. polygyrus*–infected transgene negative (−ive) and positive (+ive) mice was determined by flow cytometry. (**d**) Fecal parasite egg counts were determined at day 14 postinfection and (**e**) intestinal adult worm burden enumerated at day 28 postinfection in *H. polygyrus* in Treg-replete and -depleted mice. MLN from day 7 postinfection were re-stimulated with 1 μg *H. polygyrus* excretory/secretory antigen (HES) or media for 72 h and production of antigen-specific (**f**) interleukin (IL)-4, (**g**) interferon (IFN)-γ, and (**h**) IL-6 in the supernatant was assessed by enzyme-linked immunosorbent assay (ELISA). BALB/c Foxp3.LuciDTR mice were subsequently treated with 0.5 mg anti-IFN-γ or rat IgG1 isotype control (ISO) intraperitoneally on days 2, 4, and 6 postinfection in addition to DTx treatment. (**i**) MLN from day 7 postinfection were re-stimulated with 1 μg HES or media for 72 h and production of antigen-specific IFN-γ in the supernatant was assessed by ELISA. (**j**) Intestinal parasite worm burdens were enumerated at day 28 postinfection in Treg-depleted mice treated with neutralizing IFN-γ antibody or an isotype control. MLN production of (**k**) IL-4 and (**l**) IL-6 in response to HES was measured in the same conditions described for IFN-γ. (**m**) The number of intestinal granulomas was enumerated at day 28 postinfection. (**n**) Survival of *H. polygrus*–infected transgene negative (−ive) and positive (+ive) mice was recorded. Experiments shown are one representative of two experiments with *n*⩾3 mice/group (**b**, **c**, **f**–**i**, **k**, **l**, **n**) or are pooled data fromtwo experiments with *n*⩾4 mice/group (**d**, **e**, **m**). NS, not significant.

In a complementary approach to modulate the impact of early expansion of Treg on parasite immunity, we made use of transgenic mouse models, in which diphtheria toxin receptor (DTR) and green fluorescent protein (GFP) are expressed under the Foxp3 promoter, in order to specifically deplete GFP^+^ Tregs at early time points in infection. Transgene-negative and -positive littermates of BALB/c Foxp3.LuciDTR mice were injected with 24 ng g^−1^ diphtheria toxin (DTx) at days 1, 3, and 5 in order to completely remove DTR-positive Foxp3GFP^+^ Treg populations within the MLN of transgene-positive (+ive) littermates, compared with transgene-negative (−ive) littermates by day 7 postinfection (Figure 5a,b, see Supplementary Figure S1A online). A breakthrough population of ∼3% (∼5 × 10^5^) CD4^+^Foxp3^+^GFP^−^ Tregs were detectable in the MLN of Treg-depleted mice at this time point (Figure 5c, see Supplementary Figure S1B). Surprisingly, in view of the dampened immunity in IL-2C-treated mice, the parasite egg and worm burdens of Treg-depleted mice were significantly higher at days 14 and 28 postinfection, respectively (Figure 5d,e). Although Treg depletion did not affect antigen-specific IL-4 or IL-13 production in the MLN (Figure 5f and data not shown), the production of antigen-specific IFN-γ and IL-6 was greatly increased compared with DTx-treated transgene-negative littermates (Figure 5g,h). Treg depletion also increased the activation status of Teff and breakthrough GFP^−^ Treg populations, as measured by increased CTLA-4 and CD25 expression on CD4^+^Foxp3^−^ and Foxp3^+^ cells (see Supplementary Figure S1C–I).

In view of the strong parasite-specific IFN-γ response, which accompanied greater susceptibility to infection in the Treg-depleted mice, we next administered a neutralizing Ab to IFN-γ at days 2, 4, and 6 postinfection. Although such treatment effectively reduced antigen-specific IFN-γ production in the MLN (Figure 5i), it failed to restore resistance to infection and indeed further impaired parasite immunity, as Treg-depleted mice treated with anti-IFN-γ neutralizing Ab had significantly higher worm burden at day 28 postinfection, compared with Treg-depleted mice treated with an isotype control (Figure 5j). IFN-γ neutralization resulted in compensatory increases in antigen-specific IL-4 in Treg-depleted infected mice (Figure 5k) but also further increased pro-inflammatory IL-6 (Figure 5l), a factor known to promote *H. polygyrus* persistence.^14^

Interestingly, the development of intestinal granulomas in response to infection was almost completely ablated in Treg-depleted mice (Figure 5m) and was not restored by anti-IFN-γ Ab administration, indicating that other mediators may be able to suppress granuloma formation. Despite the known role of macrophages within these granulomas,^24, 38^ we found no evidence that the proportion, proliferation, and alternative activation of PL macrophages differed between Treg-sufficient and -depleted mice (see Supplementary Figure S1I).

To test whether the increased susceptibility of BALB/c Foxp3.LuciDTR mice following Treg depletion could be reproduced in an independently generated Foxp3-DTR mouse strain, we evaluated BALB/c DEREG mice given DTx in the first 10 days of infection as previously described;^23^ however, this regimen resulted in appreciable weight loss and a high degree of morbidity so that experiments were discontinued (Figure 5n and data not shown). These data demonstrate that efficient, almost complete Treg depletion can induce immunological chaos, pathology, and severe morbidity in helminth-infected mice, which cannot be reversed by the homeostatic expansion of breakthrough populations of Treg or by administration of neutralizing Abs to inflammatory cytokines, such as IFN-γ.

### Treg depletion of C57BL/6 infected mice

**Figure 6:**
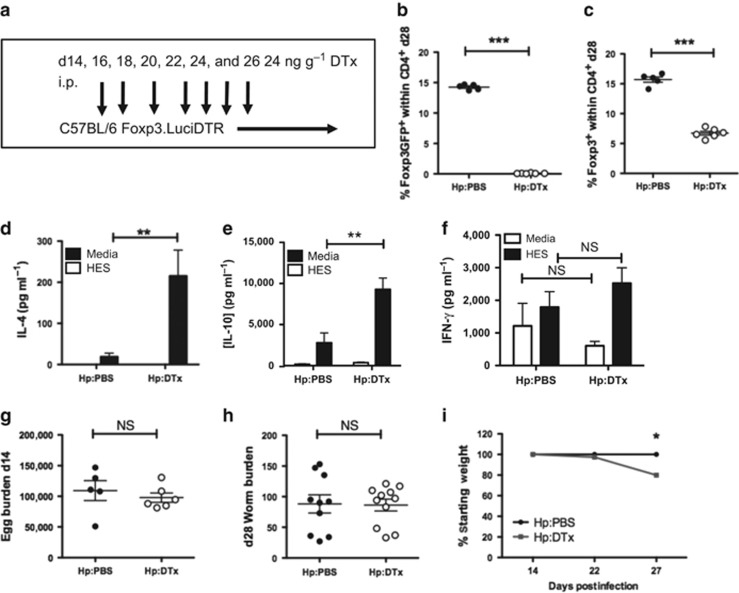
Late depletion of regulatory T cell (Treg) in more susceptible transgenic C57BL/6 Foxp3.LuciDTR mice does not alter parasite immunity. (**a**) C57BL/6 Foxp3.LuciDTR mice were treated with 24 ng g^−1^ diphtheria toxin (DTx) intraperitoneally on days 14, 16, 18, 20, 22, 24, and 26 following infection with 200 *H. polygyrus* by gavage. At day 28 postinfection, the proportion of (**b**) CD4^+^Foxp3GFP^+^ and (**c**) CD4^+^Foxp3^+^ Treg within the mesenteric lymph node (MLN) of Treg-sufficient and -depleted mice was determined by flow cytometry. MLN cells were re-stimulated with 1 μg *H. polygyrus* excretory/secretory antigen (HES) or media for 72 h and production of antigen-specific (**d**) interleukin (IL)-4, (**e**) IL-10, and (**f**) interferon (IFN)-γ assessed in supernatants by enzyme-linked immunosorbent assay. (**g**) Fecal parasite egg was determined at day 14 postinfection and (**h**) intestinal adult worm burden enumerated at day 28 postinfection in *H. polygyrus* in Treg-replete (Hp:PBS (phosphate-buffered saline)) and Treg-depleted (Hp:DTx) mice. (**i**) Body weight of Treg-replete (Hp:PBS) and Treg-depleted (Hp:DTx) mice was recorded over time following *H. polygyrus* infection as a percentage of starting weight. Experiments shown are one representative of two experiments with *n*⩾4 mice/group (**b**–**e**, **g**, **i**) or are pooled data from two experiments with *n*⩾4 mice/group (**h**) or three experiments with *n*⩾4 mice/group (**f**). NS, not significant.

Although BALB/c mice expel most parasites within 28 days, C57BL/6 mice remain susceptible to chronic infection (Figure 1a,b). To assess the impact of Treg depletion during the chronic phase of infection, we made use of transgenic Foxp3.LuciDTR mice backcrossed to the C57BL/6 background. DTx was administered every 2 days from days 14 to 26, to deplete Treg following establishment of persistent adult parasites in the murine lumen (Figure 6a). By day 28 postinfection, the Foxp3GFP^+^ population of Treg was completely depleted (Figure 6b, see Supplementary Figure S2A); however, a breakthrough population of Foxp3GFP^−^ Tregs were detectable in the MLN of Treg-depleted mice (Figure 6c, see Supplementary Figure S2B). Although Treg depletion did result in increased parasite-specific production of the Th2 cytokines IL-4, IL-13, and IL-5, as well as the regulatory cytokine IL-10, levels of IFN-γ and IL-17 remained unchanged (Figure 6d–f and data not shown). Parasite egg burden was equivalent in both groups of mice at day 14 infection before depletion (Figure 6g) and worm burden was similarly equivalent in Treg-replete and -depleted mice at day 28 postinfection (Figure 6h). There was no impact of Treg depletion on mortality, although there was an appreciable loss of body weight in the Treg-depleted group (Figure 6i). These data demonstrate that depletion of Treg late in infection does not impact on parasite burden in susceptible strains, suggesting either or both that Tregs are most critical at early stages and/or that in chronic infection multiple immunoregulatory populations such as B regulatory cells^39^ are at play.

### Partial Treg depletion with anti-CD25 Ab permits immunity to infection

**Figure 7:**
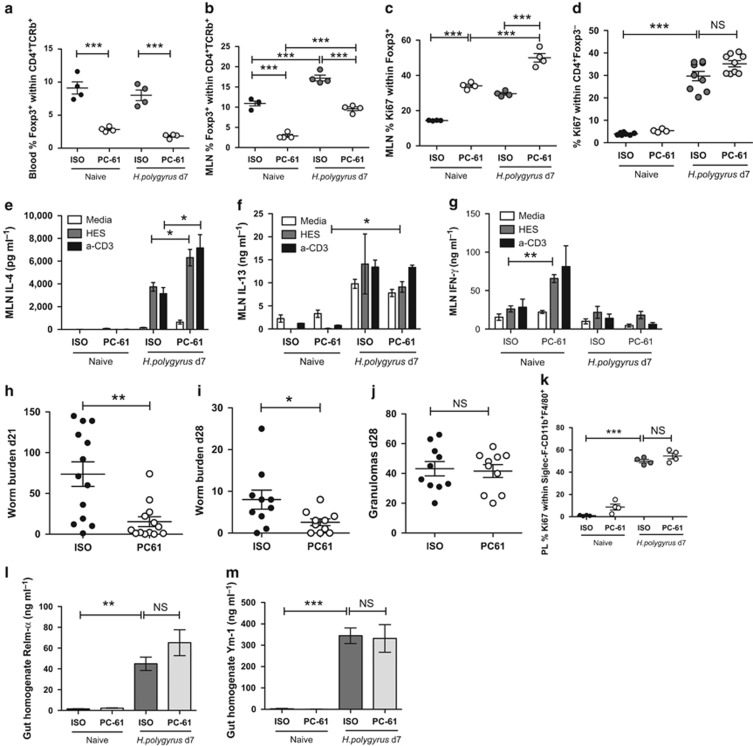
Antibody depletion of regulatory T cell (Treg) populations *in vivo* using anti-CD25 (PC61) increases parasite immunity in more resistant BALB/c mice. Naive BALB/c mice were treated with 1 mg anti-CD25 (clone PC-61) or rat IgG control (ISO) immediately before infection with 200 *H. polgyrus* by gavage. Mice were harvested at day 7 postinfection and single-cell suspensions of (**a**) blood and (**b**–**d**) mesenteric lymph node (MLN) analyzed for the proportion and proliferation (Ki67^+^) of CD4^+^Foxp3^+^ Treg and CD4^+^Foxp3^−^ T cells by flow cytometry. (**e**–**g**) MLN cells from day 7 postinfection were plated and re-stimulated with 1 μg *H. polygyrus* excretory/secretory antigen (HES), anti-CD3, or media for 72 h and production of interleukin (IL)-4, IL-13, and interferon (IFN)-γ in the supernatant was assessed by enzyme-linked immunosorbent assay. Intestinal adult worm burden was enumerated at (**h**) day 21 and (**i**) day 28 postinfection. (**j**) The number of intestinal granulomas was also enumerated at day 28 postinfection. (**k**) Macrophage proliferation (Ki67^+^) within the peritoneal lavage (PL) was determined by flow cytometry at day 7 postinfection and the production of the alternative activation markers (**l**) RELM-α and (**m**) Ym-1 within the gut homogenate determined at day 28 postinfection. Experiments shown are one representative of two experiments with *n*⩾4 mice/group (**a**–**c**, **e**, **f**, **k**–**m**) or are pooled data from two experiments with *n*⩾4 mice/group (**d**, **g**–**j**). NS, not significant.

Finally, because of the severe morbidity associated with the full depletion of Tregs in *H. polygyrus*–infected transgenic mice, we made use of a depleting Ab to CD25 (clone PC-61) to partially deplete Tregs in infected wild-type BALB/c mice. One single administration of 1 mg depleting Ab immediately before infection resulted in 70% depletion of CD4^+^Foxp3^+^ Tregs in the blood at steady state and following infection (Figure 7a). In the MLN, depletion was also around 70% at steady state but decreased to around 45% following infection (Figure 7b), which was reflected by a smaller increase in proliferation of breakthrough Treg populations in Treg-depleted, infected mice (50% Ki67^+^) (Figure 7c) compared with BALB/c Luci-DTR mice (see Supplementary Figure S1B). The number of Treg was similarly significantly reduced in the MLN of naive and *H. polygyrus*–infected mice following administration of anti-CD25 (see Supplementary Figure S2C). CD4^+^Foxp3^−^ proliferation was unaffected by anti-CD25 treatment in naive and *H. polygyrus*–infected mice (Figure 7d). This inefficient Treg depletion resulted in increased production of total IL-4 and IL-10, as well as antigen-specific IL-4 (Figure 7e and data not shown). Increased levels of IL-13 in *H. polygyrus*–infected mice were not altered by PC-61 administration (Figure 7f), neither was antigen-specific or total IFN-γ production (Figure 7g). Treg depletion resulted in the increased expulsion of adult worms at days 21 (Figure 7h) and 28 (Figure 7i) postinfection. Intestinal granuloma formation was not affected by Ab depletion of Treg in infected mice (Figure 7j) nor was the proliferation of PL macrophages (Figure 7k) or the production of RELM-α and Ym-1 in gut homogenate (Figure 7l,m). These results demonstrate that early tTreg expansion in response to chronic parasite infection is a general determinant of subsequent parasite immunity and that this response limits inflammation-induced pathology *in vivo*.

## DISCUSSION

The immune response to infection must evoke both effector and regulatory mechanisms if the reaction is to be proportionate and appropriate to the pathogen challenge. Although the role of Tregs in restraining protective immunity has been widely discussed,^8, 9^ there are fewer examples of how a measured degree of regulatory function may be necessary for the expression of functional immunity to infection.^40, 41, 42^ Here we have examined the role of Foxp3^+^ Treg populations during chronic infection in mice with the helminth *H. polygyrus*.

Early Treg expansion is evident following a number of chronic parasitic helminth infections, *Strongyloides ratti*,^43^ *Brugia malayi*,^44^ and *Litosomoides sigmodontis*,^17^ and an increase in Treg numbers correlates with increased survival of specific *T. muris* isolates.^20^ Expanded Treg numbers are also evident in mice following *H. polygyrus* infection^10, 11, 24^ with, as we now demonstrate, a marked selectivity for expansion of Helios^+^Foxp3^+^ Tregs in BALB/c mice. Helios is a marker of tTregs,^36^ which, while inducible in other T cell populations,^45, 46^ is known to synergize with Foxp3 in silencing the IL-2 locus.^47^ Our data demonstrate that Helios^+^Foxp3^+^ tTregs are the predominant population that increases in proportion and proliferation in response to infection in the more resistant BALB/c mouse strain, although, as previously demonstrated, pTreg induction can also occur.^12^ A key question was therefore to identify whether Treg expansion following *H. polygyrus* infection contributed to the parasite immune response and immune pathology in BALB/c mice.

By making use of a complex of rIL-2:anti-IL-2 (IL-2C), previously shown to specifically expand Tregs *in vivo*,^37, 48, 49^ we demonstrate that boosting of CD4^+^Helios^+^Foxp3^+^ tTreg populations early following chronic helminth infection can dampen innate and adaptive type-2 responses and decrease worm expulsion, despite increasing the number of ILCs in the MLN. Our data contribute to the hypothesis that early Treg expansion in response to chronic parasite infection is a general determinant of susceptibility to infection^17, 43, 50, 51^ and that by repressing CD4^+^ T-cell type-2 cytokine production downstream effector populations such as alternatively activated macrophages are inhibited. This pathway would be consistent with a previous report in which administration of IL-2C induced fully functional Tregs that suppressed effector CD4^+^ T-cell proliferation and IL-5^+^ and IL-13^+^ production in airway inflammation.^48^ Therefore, tTreg expansion may also limit early antigen-specific and bystander type-2 responses in a number of Th2 inflammatory settings.

Administration of IL-2C also inhibited macrophage proliferation, expression of markers of alternative activation, and granuloma formation in response to *H. polygyrus* infection, while simultaneously increasing the proportion of ILC populations *in vivo*. Macrophages have previously been shown to regulate intestinal homeostasis and enhance Treg accumulation in tissues;^52, 53^ however, boosting of Treg populations with IL-2C reduced macrophage accumulation within lesions in a murine model of atherosclerosis.^54^ Macrophage proliferation and alternative activation in response to helminth infection is reliant on local production of IL-4 and CSF-1 and requires macrophage-intrinsic IL-4Rα signaling.^55^ As IL-4 production from the adaptive immune system is required to sustain macrophage accumulation following nematode infection,^56^ our results would support the hypothesis that Treg expansion acts indirectly on macrophage populations by inhibiting adaptive type-2 cytokine responses.

The complex of rIL-2 and anti-IL-2 (JES6-1) selectively stimulate cells expressing high levels of CD25^+^, including not only Tregs but also ILC2 cells which were found in greater numbers in the MLN following IL-2C administration. CD25 is expressed at high levels by a subset of IL-5^+^ and IL-13^+^ ILC2 cells within the lung and mediastinal lymph node of naive mice.^57^ Interestingly, despite expanded ILC populations in IL-2C-treated mice, we noted that infected BALB/c mice had higher adult worm burdens and were more susceptible to parasite infection, arguing that ILC-derived cytokine production is insufficient to drive immunity. These data add to our recent findings that IL-25-induced stimulation of ILC populations in early *H. polygyrus* infection does not induce worm expulsion, suggesting that ILC2s do not have a critical role in determining immunity to this parasite (unpublished data).

We also noted a significant reduction in the proliferation of CD4^+^Foxp3^−^ cells in IL-2C-treated mice, commensurate with a reduction in the total levels of regulatory and Th2 cytokines, suggesting that an increase in activated CD25^+^CD4^+^ effector T cells does not contribute to the phenotype of *H. polygyrus*–infected IL-2C-treated mice. As a result of diminished effector T-cell proliferation following IL-2C treatment, total lymph node populations are less numerous in these mice, although numbers of Tregs are similar to the control animals. The greater susceptibility of IL-2C-treated mice therefore argues that a higher Treg:Teff ratio and/or the ability of IL-2C to activate Tregs are more critical to the outcome of infection than the absolute number of Tregs. The IL-2 complex has also been reported to selectively expand naive antigen-specific CD8^+^ T cells *in vitro* and *in vivo*.^58^ However, we recently demonstrated that immunity to *H. polygyrus* is not significantly influenced by CD8^+^ T cells,^24^ indicating that any effect of IL-2C on antigen-specific CD8^+^ T-cell expansion is unlikely to impact on helminth immunity in this model.

By making use of transgenic BALB/c Foxp3.LuciDTR mice, we found that near-complete depletion of Treg during the early phases of helminth infection caused profound immune disruption, with increased pro-inflammatory cytokine production and the extensive homeostatic expansion and outgrowth of remaining Treg, similar to a previous report on tumor-bearing mice with this transgene.^59^ During the published characterization of naive Foxp3.LuciDTR-4 mice, Treg depletion was accompanied by increased activation of conventional CD4^+^Foxp3^−^ T cells and homeostatic expansion of functional Tregs, promoted by dendritic cells.^26^ Treg depletion during *H. polygyrus* infection also resulted in significant increases in CD4^+^ T-cell activation, as indicated by upregulation of CTLA-4 and CD25 on CD4^+^Foxp3^−^ and GFP-negative Foxp3^+^ T cells. Depletion also resulted in a significant outgrowth of Foxp3^+^GFP^−^ Treg and an increase in the production of the pro-inflammatory cytokines IFN-γ and IL-6 in *H. polygyrus*–infected mice. Given the established role of dendritic cells in driving CD4^+^ T-cell activation and pro-inflammatory cytokine production, it is likely that Treg control of dendritic cell function underlies the subsequent control of parasite immunity.^60^ Near-complete depletion of Tregs in an independently constructed transgenic mouse, the BALB/c DEREG, at days 1, 3, and 5 following infection with *H. polygyrus* and in the BALB/c Foxp3.LuciDTR at days 4, 6, 8, and 10 postinfection (similar to Rausch *et al.*^23^) resulted in weight loss and a high severity score, so that experiments were discontinued. Interestingly, a previous report suggests that Tregs returning after depletion in DEREG mice are not functional in an *in vitro*-suppression assay,^61^ which may explain the pathology we see in these transgenic mice. Heightened pathology in BALB/c DEREG mice may also be explained by an increased overall dose of DTx given to these mice (1 μg vs. ∼480 ng for BALB/c Foxp3.LuciDTR), supporting the conclusions of a recent publication reporting pathology in DTx-treated wild-type mice following influenza infection.^62, 63^ Furthermore, near-complete depletion of Tregs in more susceptible C57BL/6 Foxp3.LuciDTR at days 14–26 every 2 days following infection with *H. polygyrus* resulted in weight loss but no change to parasite burden, despite increased antigen-specific Th2 responses in Treg-depleted mice. These experiments clearly demonstrate a role for Treg in limiting inflammation-induced pathology following helminth infection, as well as redundancy for Treg in controlling parasite immunity during chronicity.

Tregs have a complex role in regulating pathology in response to helminth infection, inhibiting the development of colonic granulomas during schistosomiasis^64^ in response to Toll-like receptor 2 ligation^18^ but both diminishing and promoting pathology following *T. muris* infection, depending on early or late depletion, respectively.^21^ Pathology also occurred following depletion of Tregs at days 4, 6, 8, and 10 in *H. polygyrus*–infected C57BL/6 DEREG mice, as the adult worm emerges into the lumen, which was characterized by villous blunting and atrophy, crypt hyperplasia and formation, or cellular infiltrate within the LP.^23^ This response was associated with increased antigen-specific IFN-γ and Th2 cytokine production in the MLN. IFN-γ production has been associated with increased epithelial cell proliferation and cecal crypt hyperplasia during *T. muris* infection^65^ and, moreover, with lymphedema in filariasis patients lacking Tregs,^66^ suggesting that pathology we observed in infected Treg-depleted BALB/c Foxp3.LuciDTR mice may also involve IFN-γ as well as other factors. Antigen-specific IFN-γ production is dramatically increased in infected Treg-depleted BALB/c Foxp3.LuciDTR mice; however, adult worm burden, IL-6, and IL-4 cytokine production is significantly increased on neutralization of IFN-γ in these mice. Neutralization of IFN-γ in Treg-depleted BALB/c Foxp3.LuciDTR had no impact on granuloma formation, Teff, or Treg activation status; however, it did heighten the percentage of macrophages, perhaps by allowing IL-4 to dominate.^55^ The Th2 cytokine IL-13 also has an established role in enhancing liver fibrosis and pulmonary granuloma formation following exposure to *S. mansoni* eggs^67, 68^ and is also associated with pathology in patients with ulcerative colitis.^69^ Although we found no increase in antigen-specific IL-13 following depletion of Tregs in *H. polygyrus*–infected BALB/c Foxp3.LuciDTR mice, it remains to be determined whether Treg expansion following chronic parasite infection can influence IL-13 production from populations of innate intraepithelial natural killer cells and LP natural killer T cells, known to have a role in driving pathology following *Trichinella spiralis* infection and in human ulcerative colitis.^70, 71^

Parasite pathology can further be regulated by stimulation of macrophages, which have a fundamental role in promoting intestinal tolerance and wound healing.^72, 73^ Although we observed that boosting Treg populations *in vivo* can inhibit macrophage proliferation and alternative activation following *H. polygyrus* infection, we do not observe differences in macrophage proliferation and alternative activation in infected BALB/c Foxp3.LuciDTR mice depleted or replete of Tregs, despite a loss of intestinal granulomas in Treg-depleted mice. Recently, intestinal granulomas were found to form in response to exposure to commensal flora following *S. mansoni* infection.^74^ Furthermore, colonization of wild-type mice with *Clostridium* or *Lactobacillus* commensal species was able to promote Treg differentiation or Treg activity in the intestine.^75, 76^ *H. polygyrus* has a profound impact on the microflora of the host, significantly increasing the abundance of lactobacillaceae family within the ileum of infected mice,^77^ where duodenal *Lactobacillus/Lactococcus* abundance positively correlated with the total number of Foxp3^+^CD4^+^ Tregs within the MLN of *H. polygyrus*–infected mice.^31^ Therefore, near-complete Treg depletion may alter the host response to increased *Lactobacillus/Lactococcus* abundance following *H. polygyrus* infection and result in the disrupted granuloma formation and pro-inflammatory cytokine production seen here.

Finally, we made use of Abs to CD25 to partially remove Treg populations during the early phases of *H. polygyrus* infection, which increased antigen-specific and polyclonal Th2 responses and adult worm expulsion in BALB/c mice, consistent with our hypothesis that Treg control of CD4^+^ T-cell cytokine release is instrumental in inhibiting immunity. Although anti-CD25 could also deplete activated effector populations, loss of such cells cannot account for heightened immunity we observed following Ab treatment. Partial depletion of Tregs using CD25 has also been shown to enhance IL-4 production in *S. mansoni* egg-induced inflammation^78^ and increase Th2 responses following muscle infection with *T. spiralis*.^79^ Further, combined treatment with CD25 and GITR Abs enhanced antigen-specific Th2 responses and filarial clearance in *L. sigmodontis*–infected mice,^15^ therefore our data add to the consensus that early Treg expansion can limit the antiparasite response.

In adult mice, and in the absence of an infectious challenge, inflammatory disorders such as autoimmunity do not develop following Treg depletion. However, in the presence of mutated Foxp3 alleles, such as the Foxp3(GFP)knock-in DEREG mice,^80^ CNS-1(GFP) knock-in,^5^ or *scurfy* × DEREG cross,^81^ Th2 inflammation and pathology ensue. Given that we observed severe pathology in Treg-depleted *H. polygyrus*–infected DEREG or Foxp3.LuciDTR mice, but not in anti-CD25-treated mice, a residual level of Tregs expressing wild-type Foxp3 alleles may be necessary to suppress excessive inflammation-associated pathology in this setting. Low levels of Tregs may also contribute to intestinal homeostasis and control inflammation by limiting a breach of the intestinal barrier by microbiota.^82^

Another key cytokine for controlling parasite-related pathology and regulating Th1 and Th2 inflammatory cytokine production is IL-10,^83, 84^ which is upregulated by both CD4^+^Foxp3^−^ and CD4^+^Foxp3^+^ subsets following *H. polygyrus* infection.^13^ Our findings of decreased IL-10 following IL-2C treatment and increased IL-10 following Treg depletion reveal that it is unlikely that IL-10 from CD4^+^Foxp3^−^ cells regulates inflammatory responses following infection with *H. polygyrus*. Following *T. spiralis*, *T. muris*, or *S. mansoni* infection, Foxp3^+^IL-10^−^ Tregs can restrain Th2 responses,^20, 78, 79^ whereas IL-10^+^Foxp3^+^ Treg are thought to have more of a role in controlling inflammation-induced pathology following *S. mansoni* infection.^85, 86^ Hence, Tregs are unlikely to repress Th2 cytokine production through IL-10, suggesting that inhibition is mediated by other suppressive cytokines (such as transforming growth factor-β) or by contact-dependent suppression through checkpoint inhibitory receptors.

Treg-derived IL-10 may neverthless be important in controlling pathology, as the elevation of multiple pro-inflammatory cytokines following Treg depletion in BALB/c Foxp3.LuciDTR mice could result from the complete loss of Treg-derived IL-10 in the intestinal tissues. This hypothesis is supported by the high mortality rates we see in BALB/c DEREG mice following Treg depletion during infection with *H. polygyrus* or as reported elsewhere for *S. mansoni*.^19^ Partial depletion of Foxp3^+^IL-10^+^ Treg following treatment of *H. polygyrus*–infected BALB/c mice with anti-CD25, reported at levels of around 50% at steady state within the lung tissue,^87^ would limit this response.

**Figure 8:**
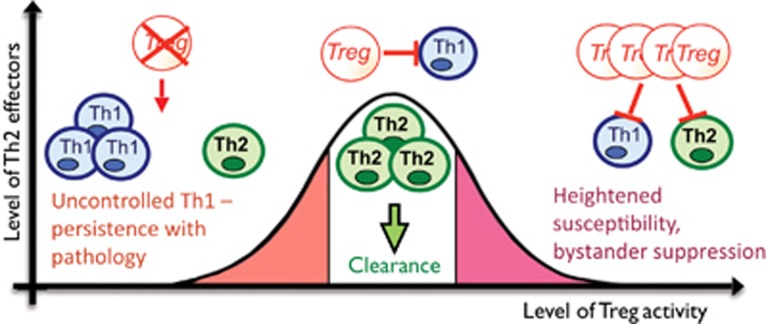
Schematic summarizing the role of regulatory T cell (Treg) in controlling pathology and inflammatory responses following *H. polygyrus* infection. Left: In *H. polygyrus* infection of mice, an absence of Treg results in uncontrolled T helper type 1 (Th1) responses and increased parasite persistence and pathology, without affecting Th2 development (e.g., in DEREG and Fop3.LuciDTR mice). Center: A low level of Treg controls excessive Th1 responses but allows Th2 responses to dominate resulting in adult worm clearance (e.g., anti-CD25 treatment). Right of the panel: A high level of Tregs (e.g., following administration of recombinant interleukin-2 (rIL-2):anti-IL-2) suppresses both Th1 and Th2 responses, increasing parasite survival and resulting in suppression of immunity to bystander antigens.

In conclusion, these data demonstrate that the presence of a low level of functional Tregs is essential not only to constrain pathology in the response to *H. polygyrus* infection but also to maintain sufficient homeostatic control of inflammatory reactions to permit a coherent protective type-2 response to evolve (Figure 8). It will be interesting to establish whether a similar non-linear relationship between the regulatory and effector T-cell compartments is in play with other helminth infections and, if so, to develop strategies to modulate Treg activity in a measured and calibrated manner to boost protective immunity.

## METHODS

**Mice.** BALB/c and C57BL/6 mice were bred in house under specific pathogen-free conditions. Foxp3.LuciDTR-4 mice previously described^26, 59^ were re-derived on a BALB/c and C57BL/6 background at the University of Edinburgh. BALB/c DEREG mice were also used as described.^88^ All protocols were approved by the University of Edinburgh's Ethical Review Committee and all animal work was conducted under UK Home Office licence. DTx was administered intraperitoneally (i.p.) to Foxp3GFP-negative and -positive littermates at a dose of 24 ng g^−1^ for Foxp3.LuciDTR-4 mice and 1 μg per animal (approximately 40 ng g^−1^) for DEREG mice, unless otherwise indicated. Mice were monitored for weight loss, physical, and behavioral changes and were euthanized if they lost >20% body weight or were otherwise deemed to have developed severe pathology.

**Parasites and antigens.** *H. polygyrus* (*bakeri*) was maintained as described elsewhere.^89^ *H. polygyrus* excretory/secretory antigen (HES) was collected from adult worms as previously detailed.^12^ Mice were infected with 200 *H. polygyrus* L3 larvae using a gavage tube. Parasite egg burden was calculated per gram of fecal pellet, and intestinal worm burden was enumerated visually at postmortem.

**In vivo Ab depletion and administration of rIL-2:anti-IL-2 (IL-2C).** Mice received 1 mg of anti-CD25 mAb (PC-61, in house) or rat immunoglobulin G (IgG; in house) i.p. immediately before infection. For rIL-2:anti-IL-2 (IL-2C) administration, mice received 2.5 μg rIL-2 (eBioscience, Hatfield, UK) with 25 μg anti-IL-2 (clone JES6-1A12; eBioscience) or 25μg rat IgG2a alone (eBioscience) i.p. Ab complex was incubated at room temperature for 30 min and administered immediately before infection. Neutralizing anti-IFN-γ (clone XMG1.2) or a rat IgG1 isotype control (clone GL113; both in house) were administered i.p. at 0.5 mg per animal.

**Abs and reagents.** Ab pairs used for cytokine enzyme-linked immunosorbent assay were as follows: IL-4 (11B11/BVD6-24G2), IL-10 (JES5–16E3/JES5-2A5), IL-13 (eBio13A/eBio1316H), IL-6 (MP5-20F3/MP5-32C11), IL-17 (eBio17B7/eBio17CK15A5), all eBioscience; and IFN-γ (R4-6A2/XMG1.2; BD Pharmingen, Oxford, UK). RELMα content was measured using the Ab pair clone 22603 and BAF1523 and Ym-1 using the mouse chitinase 3-like 3/ECF-L Duoset (both R&D, Abingdon, UK). Biotin detection Abs were used with ExtrAvadin-alkaline phosphatase conjugate (Sigma-Aldrich, Dorset, UK) and SIGMAFAST p-Nitrophenyl phosphate substrate (Sigma-Aldrich). For cell surface flow cytometry, a combination of the following Abs were applied: CD4 (RM4-5 or GK1.5; Biolegend, London, UK), CD8 (53-6.7; Biolegend), CD3 (17A2; Biolegend), CD5 (53-7.3; Biolegend), CD11c (N418; Biolegend), CD19 (6D5; Biolegend), CD25 (7D4 or PC-61; BD Pharmingen), Siglec-F (E50–2440; BD Pharmingen), CD11b (M1/70; Biolegend), F4/80 (BM8; Biolegend), ICOS (15F9; eBioscience), CD49b (DX-5; Biolegend), and CD103 (M290; BD Pharmingen). Vβ usage was assessed using a mouse TCR Vβ screening panel (BD Pharmingen). For intracellular staining, the following Abs were applied using the eBioscience Foxp3 Permabilization Kit: Ki-67 (B56; BD Pharmingen), RELMα (226033; R&D, and rabbit IgG AF647 Labeling Kit; Invitrogen, Paisley, UK), Foxp3 (FJK-16s; eBioscience), Helios (22F6; Biolegend), and CTLA-4 (UC10-4F10–11; BD Pharmingen). Staining was compared with the relevant isotype control, and acquisition was performed using an LSR 2 (BD Biosciences, Oxford, UK) and a FACSCanto (BD Biosciences). Data were analyzed using the Flowjo software (Treestar, Ashland, OR).

**Immunological assays.** Treg depletion was monitored by fluorescence-activated cell sorting analysis of peripheral (cheek) blood or MLN. Fluorescence-activated cell sorting analysis of Treg proportions in the LP extracted from the duodenum to the apex of the cecum was also performed following isolation using liberase digestion. Briefly, the intestine was removed of Peyer's Patches and intraepithelial lymphocytes were stripped by three sequential 30-s washes using media containing 2 mM EDTA. Digest of the LP was then performed for two 12-min incubations using 1 mg Liberase TL (Roche, Burgess Hill, UK) and 5 mg deoxyribonuclease I from bovine pancreas; Sigma-Aldrich). Single-cell suspensions were recovered following homogenization through a 70-and 40-μM cell strainer. Peritoneal exudate cells were collected by flushing the peritoneal cavity using 2 × 5 ml RPMI. Gut homogenate was prepared by homogenization of 1 cm of the apical duodenum in 1 × lysis buffer (Cell Signaling, Hertfordshire, UK) containing 1/100 phenylmethanesulfonylfluoride (Sigma-Aldrich) by TissueLyser (Qiagen, Manchester, UK), and the supernatant was assessed for factors associated with alternative activation by enzyme-linked immunosorbent assay.

MLN were also used for analysis of intracellular cytokine staining and antigen-specific restimulation. Briefly, 5 × 10^6^ MLN cells were stimulated with 0.5 μg phorbol 12-myristate 13-acetate and 1 μg Ionomycin (both Sigma-Aldrich) for 1 h at 37 °C/4% CO_2_ before addition of 10 μg Brefeldin A (Sigma-Aldrich) for a further 2.5 h. Cells were harvested, blocked with 2.5 μg rat IgG and stained for cell surface and intracellular markers using combinations of the Abs listed above. For antigen-specific restimulation, 5 × 10^5^ cells were plated in duplicate with media or 1 μg HES for 72 h at 37 °C/4% CO_2_. Supernatants were harvested and analyzed for cytokine production by enzyme-linked immunosorbent assay, using the Ab pairs described above.

**Statistics.** Data were assessed for normality and equal variance and were log transformed if required using the GraphPad Prism software (La Jolla, CA). For comparison between two groups, an unpaired *T*-test was used; where more than three groups were being tested, a parametric one-way analysis of variance with Tukey's multiple comparison was used. For correlation analyses on non-parametric data, a Spearman correlation test was used. The correlation co-efficient *r* value was added to graphs where correlations reached significance. NS on graphs denotes no statistical differences, *P⩽0.05, **P⩽0.01, and ***P⩽0.001.

## Notes

The authors declared no conflict of interest.

## References

[bib1] Sakaguchi, S., Yamaguchi, T., Nomura, T. & Ono, M. Regulatory T cells and immune tolerance. Cell 133, 775–787 (2008). 18510923 10.1016/j.cell.2008.05.009

[bib2] Rudensky, A.Y. Regulatory T cells and Foxp3. Immunol. Rev. 241, 260–268 (2011). 21488902 10.1111/j.1600-065X.2011.01018.x PMC3077798

[bib3] Shevach, E.M. Biological functions of regulatory T cells. Adv. Immunol. 112, 137–176 (2011). 22118408 10.1016/B978-0-12-387827-4.00004-8

[bib4] Barnes, M.J. & Powrie, F. Regulatory T cells reinforce intestinal homeostasis. Immunity 31, 401–411 (2009). 19766083 10.1016/j.immuni.2009.08.011

[bib5] Josefowicz, S.Z. et al. Extrathymically generated regulatory T cells control mucosal Th2 inflammation. Nature 482, 395–399 (2012). 22318520 10.1038/nature10772 PMC3485072

[bib6] Ai, T.L., Solomon, B.D. & Hsieh, C.S. T-cell selection and intestinal homeostasis. Immunol. Rev. 259, 60–74 (2014). 24712459 10.1111/imr.12171 PMC4028094

[bib7] Barbi, J., Pardoll, D. & Pan, F. Treg functional stability and its responsiveness to the microenvironment. Immunol. Rev. 259, 115–139 (2014). 24712463 10.1111/imr.12172 PMC3996455

[bib8] Belkaid, Y. & Tarbell, K. Regulatory T cells in the control of host-microorganism interactions. Annu. Rev. Immunol. 27, 551–589 (2009). 19302048 10.1146/annurev.immunol.021908.132723

[bib9] Maizels, R.M. & Smith, K.A. Regulatory T cells in infection. Adv. Immunol. 112, 73–136 (2011). 22118407 10.1016/B978-0-12-387827-4.00003-6 PMC7150045

[bib10] Finney, C.A.M., Taylor, M.D., Wilson, M.S. & Maizels, R.M. Expansion and activation of CD4^+^CD25^+^ regulatory T cells in *Heligmosomoides polygyrus* infection. Eur. J. Immunol. 37, 1874–1886 (2007). 17563918 10.1002/eji.200636751 PMC2699425

[bib11] Rausch, S. et al. Functional analysis of effector and regulatory T cells in a parasitic nematode infection. Infect. Immun. 76, 1908–1919 (2008). 18316386 10.1128/IAI.01233-07 PMC2346705

[bib12] Grainger, J.R. et al. Helminth secretions induce *de novo* T cell Foxp3 expression and regulatory function through the TGF-β pathway. J. Exp. Med. 207, 2331–2341 (2010). 20876311 10.1084/jem.20101074 PMC2964568

[bib13] Redpath, S.A. et al. ICOS controls Foxp3^+^ regulatory T cell expansion, maintenance, and IL-10 production during helminth infection. Eur. J. Immunol. 43, 705–715 (2013). 23319295 10.1002/eji.201242794 PMC3615169

[bib14] Smith, K.A. & Maizels, R.M. IL-6 controls susceptibility to helminth infection by impeding Th2 responsiveness and altering the Treg phenotype in vivo. Eur. J. Immunol. 44, 150–161 (2014). 24185641 10.1002/eji.201343746 PMC3992848

[bib15] Taylor, M., Le Goff, L., Harris, A., Malone, E., Allen, J.E. & Maizels, R.M. Removal of regulatory T cell activity reverses hyporesponsiveness and leads to filarial parasite clearance in vivo. J. Immunol. 174, 4924–4933 (2005). 15814720 10.4049/jimmunol.174.8.4924

[bib16] Taylor, M.D. et al. CTLA-4^+^ and CD4^+^CD25^+^ regulatory T cells inhibit protective immunity to filarial parasites in vivo. J. Immunol. 179, 4626–4634 (2007). 17878360 10.4049/jimmunol.179.7.4626

[bib17] Taylor, M.D. et al. Early recruitment of natural CD4^+^Foxp3^+^ regulatory T cells by infective larvae determines the outcome of filarial infection. Eur. J. Immunol. 39, 192–206 (2009). 19089814 10.1002/eji.200838727

[bib18] Layland, L.E., Rad, R., Wagner, H. & da Costa, C.U. Immunopathology in schistosomiasis is controlled by antigen-specific regulatory T cells primed in the presence of TLR2. Eur. J. Immunol. 37, 2174–2184 (2007). 17621370 10.1002/eji.200737063

[bib19] Layland, L.E. et al. Schistosoma mansoni-mediated suppression of allergic airway inflammation requires patency and Foxp3+ Treg cells. PLoS Negl. Trop. Dis. 7, e2379 (2013). 23967364 10.1371/journal.pntd.0002379 PMC3744427

[bib20] D'Elia, R., Behnke, J.M., Bradley, J.E. & Else, K.J. Regulatory T cells: a role in the control of helminth driven intestinal pathology and worm survival. J. Immunol. 182, 2340–2348 (2009). 19201888 10.4049/jimmunol.0802767 PMC2649429

[bib21] Sawant, D.V., Gravano, D.M., Vogel, P., Giacomin, P., Artis, D. & Vignali, D.A.A. Regulatory T cells limit induction of protective immunity and promote immune pathology following intestinal helminth infection. J. Immunol. 192, 2904–2912 (2014). 24532574 10.4049/jimmunol.1202502 PMC3955731

[bib22] Worthington, J.J. et al. Loss of the TGFβ-activating integrin αvβ8 on dendritic cells protects mice from chronic intestinal parasitic infection via control of type 2 immunity. PLoS Pathog. 9, e1003675 (2013). 24098124 10.1371/journal.ppat.1003675 PMC3789784

[bib23] Rausch, S. et al. Establishment of nematode infection despite increased Th2 responses and immunopathology after selective depletion of Foxp3^+^ cells. Eur. J. Immunol. 39, 3066–3077 (2009). 19750483 10.1002/eji.200939644

[bib24] Filbey, K.J. et al. Innate and adaptive type 2 immune cell responses in genetically controlled resistance to intestinal helminth infection. Immunol. Cell Biol. 92, 436–448 (2014). 24492801 10.1038/icb.2013.109 PMC4038150

[bib25] Lahl, K. et al. Selective depletion of Foxp3^+^ regulatory T cells induces a scurfy-like disease. J. Exp. Med. 204, 57–63 (2007). 17200412 10.1084/jem.20061852 PMC2118432

[bib26] Suffner, J. et al. Dendritic cells support homeostatic expansion of Foxp3^+^ regulatory T cells in Foxp3.LuciDTR mice. J. Immunol. 184, 1810–1820 (2010). 20083650 10.4049/jimmunol.0902420

[bib27] Wilson, M.S., Taylor, M., Balic, A., Finney, C.A.M., Lamb, J.R. & Maizels, R.M. Suppression of allergic airway inflammation by helminth-induced regulatory T cells. J. Exp. Med. 202, 1199–1212 (2005). 16275759 10.1084/jem.20042572 PMC2213237

[bib28] Setiawan, T. et al. *Heligmosomoides polygyrus* promotes regulatory T-cell cytokine production in the murine normal distal intestine. Infect. Immun. 75, 4655–4663 (2007). 17606601 10.1128/IAI.00358-07 PMC1951154

[bib29] Prowse, S.J., Mitchell, G.F., Ley, P.L. & Jenkin, C.R. The development of resistance in different inbred strains of mice to infection with *Nematospiroides dubius*. Parasite Immunol. 1, 277–288 (1979). 551381 10.1111/j.1365-3024.1979.tb00713.x

[bib30] Behnke, J.M., Menge, D.M. & Noyes, H. *Heligmosomoides bakeri:* a model for exploring the biology and genetics of restance to chronic gastrointestinal nematode infections. Parasitology 136, 1565–1580 (2009). 19450375 10.1017/S0031182009006003

[bib31] Reynolds, L.A. et al. Commensal-pathogen interactions in the intestinal tract: Lactobacilli promote infection with, and are promoted by, helminth parasites. Gut Microbes 5, 10–19 (2014). 10.4161/gmic.32155 PMC4822684 25144609

[bib32] Huehn, J. et al. Developmental stage, phenotype, and migration distinguish naive- and effector/memory-like CD4^+^ regulatory T cells. J. Exp. Med. 199, 303–313 (2004). 14757740 10.1084/jem.20031562 PMC2211798

[bib33] Sugimoto, N. et al. Foxp3-dependent and -independent molecules specific for CD25^+^CD4^+^ natural regulatory T cells revealed by DNA microarray analysis. Int. Immunol. 18, 1197–1209 (2006). 16772372 10.1093/intimm/dxl060

[bib34] Yadav, M. et al. Neuropilin-1 distinguishes natural and inducible regulatory T cells among regulatory T cell subsets in vivo. J. Exp. Med. 209, 1713–1722 (2012). 22966003 10.1084/jem.20120822 PMC3457729

[bib35] Weiss, J.M. et al. Neuropilin 1 is expressed on thymus-derived natural regulatory T cells, but not mucosa-generated induced Foxp3+ T reg cells. J. Exp. Med. 209, 1723–1742 (2012). 22966001 10.1084/jem.20120914 PMC3457733

[bib36] Thornton, A.M. et al. Expression of Helios, an Ikaros transcription factor family member, differentiates thymic-derived from peripherally induced Foxp3^+^ T regulatory cells. J. Immunol. 184, 3433–3441 (2010). 20181882 10.4049/jimmunol.0904028 PMC3725574

[bib37] Boyman, O., Kovar, M., Rubinstein, M.P., Surh, C.D. & Sprent, J. Selective stimulation of T cell subsets with antibody-cytokine immune complexes. Science 311, 1924–1927 (2006). 16484453 10.1126/science.1122927

[bib38] Anthony, R.M. et al. Memory T_H_2 cells induce alternatively activated macrophages to mediate protection against nematode parasites. Nat. Med. 12, 955–960 (2006). 16892038 10.1038/nm1451 PMC1955764

[bib39] Wilson, M.S. et al. Helminth-induced CD19^+^CD23^hi^ B cells modulate experimental allergic and autoimmune inflammation. Eur. J. Immunol. 40, 1682–1696 (2010). 20306466 10.1002/eji.200939721 PMC3179601

[bib40] Lund, J.M., Hsing, L., Pham, T.T. & Rudensky, A.Y. Coordination of early protective immunity to viral infection by regulatory T cells. Science 320, 1220–1224 (2008). 18436744 10.1126/science.1155209 PMC2519146

[bib41] Ruckwardt, T.J., Bonaparte, K.L., Nason, M.C. & Graham, B.S. Regulatory T cells promote early influx of CD8+ T cells in the lungs of respiratory syncytial virus-infected mice and diminish immunodominance disparities. J. Virol. 83, 3019–3028 (2009). 19153229 10.1128/JVI.00036-09 PMC2655550

[bib42] Wang, Z. et al. Regulatory T cells promote a protective Th17-associated immune response to intestinal bacterial infection with C. rodentium. Mucosal Immunol. 7, 1290–1301 (2014). 24646939 10.1038/mi.2014.17

[bib43] Blankenhaus, B. et al. *Strongyloides ratti* infection induces expansion of Foxp3^+^ regulatory T cells that interfere with immune response and parasite clearance in BALB/c mice. J. Immunol. 186, 4295–4305 (2011). 21335490 10.4049/jimmunol.1001920

[bib44] McSorley, H.J., Harcus, Y.M., Murray, J., Taylor, M.D. & Maizels, R.M. Expansion of Foxp3^+^ regulatory T cells in mice infected with the filarial parasite *Brugia malayi*. J. Immunol. 181, 6456–6466 (2008). 18941236 10.4049/jimmunol.181.9.6456

[bib45] Akimova, T., Beier, U.H., Wang, L., Levine, M.H. & Hancock, W.W. Helios expression is a marker of T cell activation and proliferation. PLoS One 6, e24226 (2011). 21918685 10.1371/journal.pone.0024226 PMC3168881

[bib46] Gottschalk, R.A., Corse, E. & Allison, J.P. Expression of Helios in peripherally induced Foxp3+ regulatory T cells. J. Immunol. 188, 976–980 (2012). 22198953 10.4049/jimmunol.1102964

[bib47] Baine, I., Basu, S., Ames, R., Sellers, R.S. & Macian, F. Helios induces epigenetic silencing of IL2 gene expression in regulatory T cells. J. Immunol. 190, 1008–1016 (2013). 23275607 10.4049/jimmunol.1200792 PMC3558938

[bib48] Wilson, M.S., Pesce, J.T., Ramalingam, T.R., Thompson, R.W., Cheever, A. & Wynn, T.A. Suppression of murine allergic airway disease by IL-2:anti-IL-2 monoclonal antibody-induced regulatory T cells. J. Immunol. 181, 6942–6954 (2008). 18981114 10.4049/jimmunol.181.10.6942 PMC2706157

[bib49] Haque, A. et al. CD4^+^ natural regulatory T cells prevent experimental cerebral malaria via CTLA-4 when expanded in vivo. PLoS Pathog. 6, e1001221 (2010). 21170302 10.1371/journal.ppat.1001221 PMC3000360

[bib50] Tang, C.L. et al. Effect of CD4^+^ CD25^+^ regulatory T cells on the immune evasion of *Schistosoma japonicum*. Parasitol. Res. 108, 477–480 (2011). 20886233 10.1007/s00436-010-2089-2

[bib51] Taylor, M.D., van der Werf, N. & Maizels, R.M. T cells in helminth infection: the regulators and the regulated. Trends Immunol. 33, 181–189 (2012). 22398370 10.1016/j.it.2012.01.001

[bib52] Hadis, U. et al. Intestinal tolerance requires gut homing and expansion of FoxP3+ regulatory T cells in the lamina propria. Immunity 34, 237–246 (2011). 21333554 10.1016/j.immuni.2011.01.016

[bib53] McBride, A., Konowich, J. & Salgame, P. Host defense and recruitment of Foxp3^+^ T regulatory cells to the lungs in chronic *Mycobacterium tuberculosis* infection requires toll-like receptor 2. PLoS Pathog. 9, e1003397 (2013). 23785280 10.1371/journal.ppat.1003397 PMC3681744

[bib54] Dinh, T.N. et al. Cytokine therapy with interleukin-2/anti-interleukin-2 monoclonal antibody complexes expands CD4+CD25+Foxp3+ regulatory T cells and attenuates development and progression of atherosclerosis. Circulation 126, 1256–1266 (2012). 22851544 10.1161/CIRCULATIONAHA.112.099044

[bib55] Jenkins, S.J. et al. IL-4 directly signals tissue-resident macrophages to proliferate beyond homeostatic levels controlled by CSF-1. J. Exp. Med. 210, 2477–2491 (2013). 24101381 10.1084/jem.20121999 PMC3804948

[bib56] Loke, P. et al. Alternative activation is an innate response to injury that requires CD4^+^ T cells to be sustained during chronic infection. J. Immunol. 179, 3926–3936 (2007). 17785830 10.4049/jimmunol.179.6.3926

[bib57] Klein Wolterink, R.G. et al. Pulmonary innate lymphoid cells are major producers of IL-5 and IL-13 in murine models of allergic asthma. Eur. J. Immunol. 42, 1106–1116 (2012). 22539286 10.1002/eji.201142018

[bib58] Tomala, J., Chmelova, H., Mrkvan, T., Rihova, B. & Kovar, M. In vivo expansion of activated naive CD8+ T cells and NK cells driven by complexes of IL-2 and anti-IL-2 monoclonal antibody as novel approach of cancer immunotherapy. J. Immunol. 183, 4904–4912 (2009). 19801515 10.4049/jimmunol.0900284

[bib59] Li, X., Kostareli, E., Suffner, J., Garbi, N. & Hämmerling, G.J. Efficient Treg depletion induces T-cell infiltration and rejection of large tumors. Eur. J. Immunol. 40, 3325–3335 (2010). 21072887 10.1002/eji.201041093

[bib60] Smith, K.A., Hochweller, K., Hämmerling, G.J., Boon, L., Macdonald, A.S. & Maizels, R.M. Chronic helminth infection mediates tolerance in vivo through dominance of CD11c^lo^ CD103^–^ DC population. J. Immunol. 186, 7098–7109 (2011). 21576507 10.4049/jimmunol.1003636 PMC4794626

[bib61] Lahl, K. & Sparwasser, T. In vivo depletion of Foxp3+ Tregs using the DEREG mouse model. Methods Mol. Biol. 707, 157–172 (2011). 21287334 10.1007/978-1-61737-979-6_10

[bib62] Christiaansen, A.F., Boggiatto, P.M. & Varga, S.M. Limitations of Foxp3^+^ Treg depletion following viral infection in DEREG mice. J. Immunol. Methods 406, 58–65 (2014). 24642426 10.1016/j.jim.2014.03.005 PMC4029878

[bib63] Mayer, C.T. et al. Advantages of Foxp3(+) regulatory T cell depletion using DEREG mice. Immun. Inflamm. Dis. 2, 162–165 (2014). 25505550 10.1002/iid3.33 PMC4257761

[bib64] Turner, J.D. et al. CD4^+^CD25^+^ regulatory cells contribute to the regulation of colonic Th2 granulomatous pathology caused by Schistosome infection. PLoS Negl. Trop. Dis. 5, e1269 (2011). 21858239 10.1371/journal.pntd.0001269 PMC3153428

[bib65] Artis, D., Potten, C.S., Else, K.J., Finkelman, F.D. & Grencis, R.K. *Trichuris muris*: host intestinal epithelial cell hyperproliferation during chronic infection is regulated by interferon-gamma. Exp. Parasitol. 92, 144–153 (1999). 10366539 10.1006/expr.1999.4407

[bib66] Babu, S. et al. Filarial lymphedema is characterized by antigen-specific Th1 and Th17 proinflammatory responses and a lack of regulatory T cells. PLoS Negl. Trop. Dis. 3, e420 (2009). 19381284 10.1371/journal.pntd.0000420 PMC2666805

[bib67] Chiaramonte, M.G., Schopf, L.R., Neben, T.Y., Cheever, A.W., Donaldson, D.D. & Wynn, T.A. IL-13 is a key regulatory cytokine for Th2 cell-mediated pulmonary granuloma formation and IgE responses induced by *Schistosoma mansoni* eggs. J. Immunol. 162, 920–930 (1999). 9916716

[bib68] Chiaramonte, M.G., Donaldson, D.D., Cheever, A.W. & Wynn, T.A. An IL-13 inhibitor blocks the development of hepatic fibrosis during a T-helper type 2-dominated inflammatory response. J. Clin. Invest. 104, 777–785 (1999). 10491413 10.1172/JCI7325 PMC408441

[bib69] Heller, F. et al. Interleukin-13 is the key effector Th2 cytokine in ulcerative colitis that affects epithelial tight junctions, apoptosis, and cell restitution. Gastroenterology 129, 550–564 (2005). 16083712 10.1016/j.gastro.2005.05.002

[bib70] McDermott, J.R., Humphreys, N.E., Forman, S.P., Donaldson, D.D. & Grencis, R.K. Intraepithelial NK cell-derived IL-13 induces intestinal pathology associated with nematode infection. J. Immunol. 175, 3207–3213 (2005). 16116211 10.4049/jimmunol.175.5.3207

[bib71] Fuss, I.J. et al. Nonclassical CD1d-restricted NK T cells that produce IL-13 characterize an atypical Th2 response in ulcerative colitis. J. Clin. Invest. 113, 1490–1497 (2004). 15146247 10.1172/JCI19836 PMC406524

[bib72] Chen, F. et al. An essential role for TH2-type responses in limiting acute tissue damage during experimental helminth infection. Nat. Med. 18, 260–266 (2012). 22245779 10.1038/nm.2628 PMC3274634

[bib73] Mortha, A. et al. Microbiota-dependent crosstalk between macrophages and ILC3 promotes intestinal homeostasis. Science 343, 1249288 (2014). 24625929 10.1126/science.1249288 PMC4291125

[bib74] Holzscheiter, M. et al. Lack of host gut microbiota alters immune responses and intestinal granuloma formation during schistosomiasis. Clin. Exp. Immunol. 175, 246–257 (2014). 24168057 10.1111/cei.12230 PMC3892416

[bib75] Atarashi, K. et al. Induction of colonic regulatory T cells by indigenous *Clostridium* species. Science 331, 337–341 (2011). 21205640 10.1126/science.1198469 PMC3969237

[bib76] Tanoue, T. & Honda, K. Induction of Treg cells in the mouse colonic mucosa: a central mechanism to maintain host-microbiota homeostasis. Semin. Immunol. 24, 50–57 (2012). 22172550 10.1016/j.smim.2011.11.009

[bib77] Walk, S.T., Blum, A.M., Ewing, S.A., Weinstock, J.V. & Young, V.B. Alteration of the murine gut microbiota during infection with the parasitic helminth *Heligmosomoides polygyrus*. Inflamm. Bowel Dis. 16, 1841–1849 (2010). 20848461 10.1002/ibd.21299 PMC2959136

[bib78] Baumgart, M., Tomkins, F., Leng, J. & Hesse, M. Naturally-occurring CD4^+^Foxp3^+^ regulatory T cells are an essential, IL-10-independent part of the immunoregulatory network in *Schistosoma mansoni* egg-induced inflammation. J. Immunol. 176, 5374–5387 (2006). 16622005 10.4049/jimmunol.176.9.5374

[bib79] Beiting, D.P., Gagliardo, L.F., Hesse, M., Bliss, S.K., Meskill, D. & Appleton, J.A. Coordinated control of immunity to muscle stage *Trichinella spiralis* by IL-10, regulatory T cells, and TGF-β. J. Immunol. 178, 1039–1047 (2007). 17202367 10.4049/jimmunol.178.2.1039

[bib80] Mayer, C.T. et al. Few Foxp3(+) regulatory T cells are sufficient to protect adult mice from lethal autoimmunity. Eur. J. Immunol. 44, 2990–3002 (2014). 25042334 10.1002/eji.201344315

[bib81] Lahl, K. et al. Nonfunctional regulatory T cells and defective control of Th2 cytokine production in natural scurfy mutant mice. J. Immunol. 183, 5662–5672 (2009). 19812199 10.4049/jimmunol.0803762

[bib82] Amendola, A., Butera, A., Sanchez, M., Strober, W. & Boirivant, M. Nod2 deficiency is associated with an increased mucosal immunoregulatory response to commensal microorganisms. Mucosal Immunol. 7, 391–404 (2014). 23962873 10.1038/mi.2013.58 PMC4778708

[bib83] Wynn, T.A. et al. IL-10 regulates liver pathology in acute murine schistosomiasis mansoni but is not required for immune down-modulation of chronic disease. J. Immunol. 159, 4473–4480 (1998). 9574553

[bib84] Hoffmann, K.F., Cheever, A.W. & Wynn, T.A. IL-10 and the dangers of immune polarization: excessive type 1 and type 2 cytokine responses induce distinct forms of lethal immunopathology in murine schistosomiasis. J. Immunol. 164, 6406–6416 (2000). 10843696 10.4049/jimmunol.164.12.6406

[bib85] Hesse, M. et al. The pathogenesis of schistosomiasis is controlled by cooperating IL-10-producing innate effector and regulatory T cells. J. Immunol. 172, 3157–3166 (2004). 14978122 10.4049/jimmunol.172.5.3157

[bib86] McKee, A.S. & Pearce, E.J. CD25^+^CD4^+^ cells contribute to Th2 polarization during helminth infection by suppressing Th1 response development. J. Immunol. 173, 1224–1231 (2004). 15240714 10.4049/jimmunol.173.2.1224

[bib87] McGee, H.S., Edwan, J.H. & Agrawal, D.K. Flt3-L increases CD4^+^CD25^+^Foxp3^+^ICOS^+^ cells in the lungs of cockroach-sensitized and -challenged mice. Am. J. Respir. Cell Mol. Biol. 42, 331–340 (2010). 19448155 10.1165/rcmb.2008-0397OC PMC2830405

[bib88] Baru, A.M. et al. Selective depletion of Foxp3+ Treg during sensitization phase aggravates experimental allergic airway inflammation. Eur. J. Immunol. 40, 2259–2266 (2010). 20544727 10.1002/eji.200939972

[bib89] Johnston, C.J.C. et al. Cultivation of *Heligmosomoides polygyrus*: an immunomodulatory nematode parasite and its secreted products. J. Vis. Exp. 98 (2015). 10.3791/52412 PMC4401400 25867600

