# Supplementary figures and images for: Low-level regulatory T-cell activity is essential for functional type-2 effector immunity to expel gastrointestinal helminths
Source: Mucosal Immunol. 2015 Aug 19;9(2):428–43. doi: 10.1038/mi.2015.73 (PMC4677460; doi:10.1038/mi.2015.73)

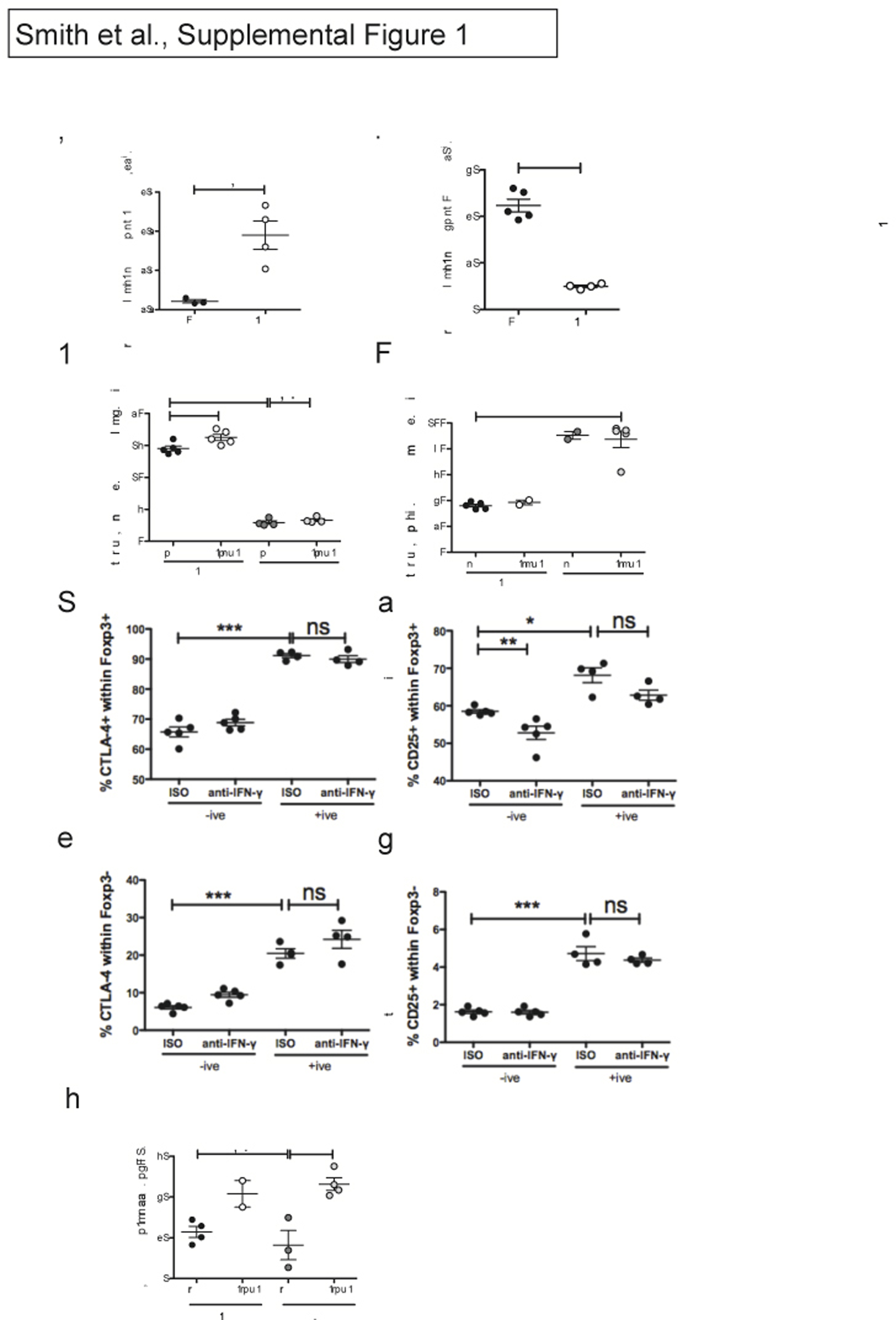

Supplement: Supplementary Figure S1 [file mi201573x1.tif]

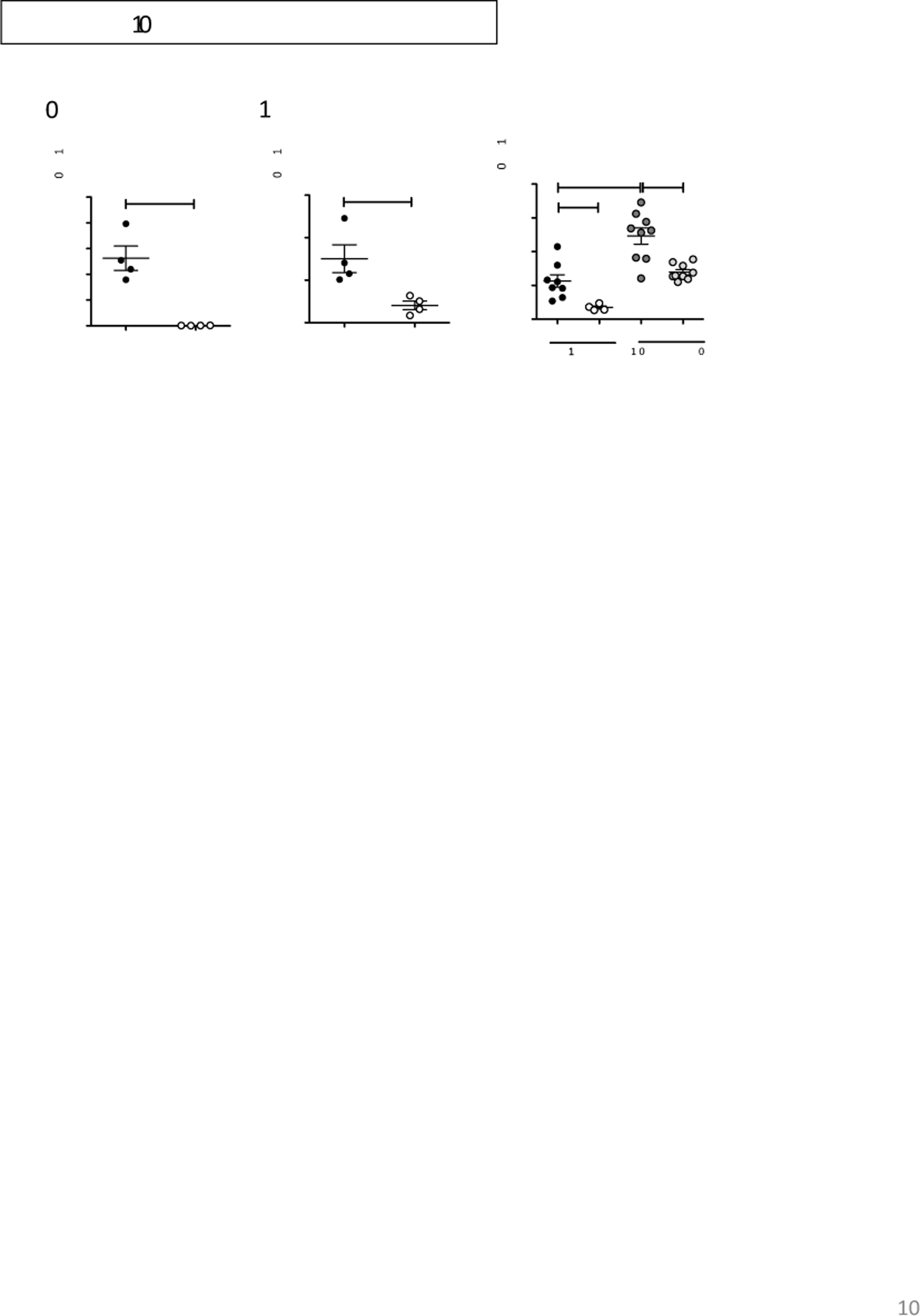

Supplement: Supplementary Figure S2 [file mi201573x2.tif]
