# Supplementary material for: Low-level regulatory T-cell activity is essential for functional type-2 effector immunity to expel gastrointestinal helminths
Source: Mucosal Immunol. 2015 Aug 19;9(2):428–43. doi: 10.1038/mi.2015.73 (PMC4677460; doi:10.1038/mi.2015.73)
Supplement: Supplementary Figure Legends [file mi201573x3.doc]

**Figure Legends**

**Supplemental Figure 1. Anti-IFN-γ treatment of Treg-depleted mice**

BALB/c Foxp3.LuciDTR mice were treated with 0.5 mg anti-IFN-γ or rat IgG1 isotype control (ISO) i.p. day 2, 4 and 6 post-infection in addition to DTx treatment with 24 ng/g diphtheria toxin (DTx) following infection with 200 *H. polygyrus* by gavage. The number of CD4^+^Foxp3GFP^+^ (**A**) and CD4^+^Foxp3GFP^-^ (**B**) Treg within the MLN of DTx treated day 7 *H. polygyrus* infected transgene negative (-ive) and positive (+ive) mice was determined by flow cytometry. The proportion (**C**) and proliferation (**D**) of CD4^+^Foxp3 ^+^ Treg within the MLN of DTx treated day 7 *H. polygyrus* infected transgene negative (-ive) and positive (+ive) mice treated with anti-IFN-γ or an isotype control (ISO). CTLA-4 (**E** and **G**) and CD25 (**F** and **H**) expression within CD4^+^Foxp3^+^ Treg (**E-F**) and CD4^+^Foxp3^–^ Teff (**G-H**). The proportions of Siglec-F^–^CD11b^+^F4/80^+^ cells within the PL of the same mice were determined by flow cytometry (**I**). Experiments shown are one representative of 2 experiments with n ≥ 4 mice/group (**A-C, E-H**) or with n ≥ 2 mice/group (**D and I**).

**Supplemental Figure 2. Treg numbers for Treg manipulation experiments.**

C57BL/6 Foxp3.LuciDTR mice were treated with 24 ng/g diphtheria toxin (DTx) i.p. day 14, 16, 18, 20, 22, 24 and 26 following infection with 200 *H. polygyrus* by gavage**.** At day 28 post-infection, the number of CD4^+^Foxp3GFP^+^ (**A**) and CD4^+^Foxp3GFP^-^ Treg (**B**) within the MLN of Treg sufficient and depleted mice was determined by flow cytometry. Naïve BALB/c mice were treated with 1 mg anti-CD25 (clone PC-61) or rat IgG control (ISO) immediately before infection with 200 *H. polgyrus* by gavage. Mice were harvested at day 7 post-infection and single cell suspensions MLN analysed for the number of CD4^+^Foxp3^+^ Treg (**C**).
